# The Difficulties of Predicting the Outbreak Sizes of Epidemics

**DOI:** 10.1371/journal.pmed.0030023

**Published:** 2005-11-22

Epidemiologists have used mathematical models to predict and understand the dynamics of infectious diseases for more than 100 years. The emergence of diseases such as ebola, severe acute respiratory syndrome (SARS), West Nile virus, and multidrug-resistant malaria; incidences of bioterrorism; and most recently, the threat of a bird flu pandemic have attached even greater importance to this management tool. Models are used to provide information on the infection and to predict the effect of courses of action. The World Health Organization has said that the primary goals of any early warning system should be to predict the timing and magnitude of an outbreak. But it has said that forecasting will save the most lives when it can accurately predict the final size of the outbreak.

However, researchers admit that predicting the final size of an outbreak is notoriously difficult. For example, even for annual events such as meningitis outbreaks in West Africa, researchers still find it hard to predict the final size of the epidemic. Of course, mathematical models, whether in epidemiology or otherwise, are only as good as the assumptions on which they are based. So if a model makes predictions out of line with observed results and the calculations are correct, the initial assumptions that made the model useful must be changed.

In *PLoS Medicine*, John Drake investigates the limits of forecasting precision for directly transmitted diseases, and suggests epidemiologists shouldn't focus exclusively on the final size of an outbreak. He says the stochastic (chance) contact process by which outbreaks develop creates fundamental limits for the precision with which the final size of the outbreak can be predicted.

Drake modeled the expected final outbreak size in nine well-studied infectious diseases (chicken pox, diphtheria, measles, mumps, poliomyelitis, rubella, scarlet fever, smallpox, and whooping cough). He then applied his findings to a new model, a simple stochastic epidemic with delayed onset intervention, which represents actual outbreaks of emerging infections more realistically. He found that the final size of an outbreak is difficult to predict because of local environmental and disease-specific conditions. Also, outbreak dynamics are very susceptible to the seemingly random sequence of infectious contacts and the early removal of infectious patients from the unobserved stages of the outbreak.

The basic approach currently used by epidemiologists is to compare the average of the influencing factors with the basic reproductive ratio of the disease. This approach is fine for early warning systems, but for emerging diseases or sudden outbreaks, the final outbreak size can differ greatly from these straightforward calculations.

Drake says that a stochastic theory of epidemics, which accounts for probable changes, can better quantify whether an outbreak size can deviate from initial calculations and can account for changing removal rate and/or number of infectious contacts. He found that in epidemics the coefficient of variation in the final outbreak size was greater than one for outbreaks where the removal rate was less than about 2.41 times the contact rate. The removal rate changes when clinicians are able to increase their ability to diagnose and treat infected patients, he suggests. And, he says, the number of infectious contacts falls when the rising number of cases dilutes the remaining susceptible population. When testing these observations in a representative example, Drake found that the average outbreak size grew exponentially with the delay between the start of the outbreak and the implementation of the intervention, underscoring the importance of rapid intervention.

His findings stressed the point that rapidly starting control measures was important not only for controlling the final outbreak size but also for decreasing the variation in the final size of the outbreak. And epidemiologists should not just focus on predicting outbreak size, but also consider other characteristics, such as the timing of disease emergence.

